# Differential expression of the nuclear-encoded mitochondrial transcriptome in pediatric septic shock

**DOI:** 10.1186/s13054-014-0623-9

**Published:** 2014-11-19

**Authors:** Scott L Weiss, Natalie Z Cvijanovich, Geoffrey L Allen, Neal J Thomas, Robert J Freishtat, Nick Anas, Keith Meyer, Paul A Checchia, Thomas P Shanley, Michael T Bigham, Julie Fitzgerald, Sharon Banschbach, Eileen Beckman, Kelli Howard, Erin Frank, Kelli Harmon, Hector R Wong

**Affiliations:** Division of Critical Care Medicine, Department of Anesthesia and Critical Care, The Children’s Hospital of Philadelphia, University of Pennsylvania Perelman School of Medicine, 3620 Hamilton Walk, Philadelphia, PA 19104 USA; Center for Resuscitation Science, University of Pennsylvania Perelman School of Medicine, 3620 Hamilton Walk, Philadelphia, PA 19104 USA; UCSF Benioff Children’s Hospital Oakland, 1411 East 31st Street, Oakland, CA 94602 USA; Children’s Mercy Hospital, 2401 Gillham Road, Kansas City, MO 64108 USA; Penn State Children’s Hospital, 500 University Drive, Hershey, PA 17033 USA; Children’s National Medical Center, 111 Michigan Avenue NW, Washington, DC 20010 USA; Children’s Hospital of Orange County, 1201 W La Veta Avenue, Orange, CA 92868 USA; Miami Children’s Hospital, 3100 SW 62nd Avenue, Miami, FL 33155 USA; Texas Children’s Hospital, 6621 Fannin Street, Houston, TX 77030 USA; CS Mott Children’s Hospital at the University of Michigan, 1540 E Hospital Drive, Ann Arbor, MI 48109 USA; Akron Children’s Hospital, 1 Perkins Square, Akron, OH 44302 USA; Division of Critical Care Medicine, Cincinnati Children’s Hospital Medical Center and Cincinnati Children’s Research Foundation, 3333 Burnet Avenue, MLC 2005, Cincinnati, OH 45229 USA; Department of Pediatrics, University of Cincinnati College of Medicine, 3230 Eden Avenue, Cincinnati, OH 45267 USA

## Abstract

**Introduction:**

Increasing evidence supports a role for mitochondrial dysfunction in organ injury and immune dysregulation in sepsis. Although differential expression of mitochondrial genes in blood cells has been reported for several diseases in which bioenergetic failure is a postulated mechanism, there are no data about the blood cell mitochondrial transcriptome in pediatric sepsis.

**Methods:**

We conducted a focused analysis using a multicenter genome-wide expression database of 180 children ≤10 years of age with septic shock and 53 healthy controls. Using total RNA isolated from whole blood within 24 hours of PICU admission for septic shock, we evaluated 296 nuclear-encoded mitochondrial genes using a false discovery rate of 1%. A series of bioinformatic approaches were applied to compare differentially expressed genes across previously validated gene expression-based subclasses (groups A, B, and C) of pediatric septic shock.

**Results:**

In total, 118 genes were differentially regulated in subjects with septic shock compared to healthy controls, including 48 genes that were upregulated and 70 that were downregulated. The top scoring canonical pathway was oxidative phosphorylation, with general downregulation of the 51 genes corresponding to the electron transport system (ETS). The top two gene networks were composed primarily of mitochondrial ribosomal proteins highly connected to ETS complex I, and genes encoding for ETS complexes I, II, and IV that were highly connected to the peroxisome proliferator activated receptor (PPAR) family. There were 162 mitochondrial genes differentially regulated between groups A, B, and C. Group A, which had the highest maximum number of organ failures and mortality, exhibited a greater downregulation of mitochondrial genes compared to groups B and C.

**Conclusions:**

Based on a focused analysis of a pediatric septic shock transcriptomic database, nuclear-encoded mitochondrial genes were differentially regulated early in pediatric septic shock compared to healthy controls, as well as across genotypic and phenotypic distinct pediatric septic shock subclasses. The nuclear genome may be an important mechanism contributing to alterations in mitochondrial bioenergetic function and outcomes in pediatric sepsis.

**Electronic supplementary material:**

The online version of this article (doi:10.1186/s13054-014-0623-9) contains supplementary material, which is available to authorized users.

## Introduction

Septic shock is a leading cause of morbidity and mortality in the pediatric intensive care unit (PICU) [1,2]. With improved therapies to reverse shock, progressive multi-organ failure and secondary infection from acquired immunoparalysis are now the main antecedents to sepsis-associated death [3,4]. Increasing evidence supports a role for mitochondrial bioenergetic dysfunction in the pathobiology of organ injury and immune dysregulation in sepsis [5-7].

Circulating blood cells from critically ill patients with septic shock exhibit decreased oxidative respiration, electron chain complex activity, mitochondrial turnover, and mitochondrial membrane potential [8-12]. Blood is an easily accessible tissue that can be used to directly measure mitochondrial dysfunction in immune cells and may reflect a systemic process affecting other vital organs. Mitochondrial dysfunction in blood cells has been associated with severity of illness, organ dysfunction, mortality, and immunoparalysis in human sepsis [8-12], including children [13]. Differential expression of mitochondrial genes in blood cells has been reported for several diseases in which bioenergetic failure is a postulated mechanism [14-16], and injection of endotoxin has been shown to cause widespread suppression of genes encoding for mitochondrial ATP production and protein synthesis within human leukocytes [17]. However, there are no data about the blood cell mitochondrial transcriptome in pediatric sepsis. Identification of mitochondrial genomic changes within blood cells could provide clinically relevant biomarkers, offer insight into biological mechanisms, and inform therapeutic targets related to mitochondrial bioenergetic dysfunction for children with sepsis.

While mitochondria contain their own circular genome, the majority of the mitochondrial proteins comprising subunits of the electron transport system (ETS) are encoded by nuclear genes, including 38/45 for complex I, 4/4 for complex II, 10/11 for complex III, 10/13 for complex IV, and 17/19 for complex V (ATP synthase). In addition, all of the 79 known mitochondrial ribosomal proteins (MRPs) are encoded by the nuclear genome [18,19]. These ETS and ribosomal proteins are synthesized within the cytoplasm and then imported into the mitochondria.

Over the last decade, we have generated an extensive genome-wide expression database of children with septic shock drawn from multiple centers in the U.S. [20]. The database has enabled the discovery of gene expression-based subclasses of pediatric septic shock [21-23], stratification biomarkers [24-31], diagnostic biomarkers [32-35], and novel therapeutic targets [36-42]. Here, we mined the database to test the hypothesis that expression of whole blood-derived nuclear-encoded mitochondrial genes will be differentially regulated between pediatric patients with septic shock and nonseptic controls within the first 24 hours of presentation to the PICU. We further hypothesized that nuclear-encoded mitochondrial genes would be differentially regulated across genotypic and phenotypic distinct subclasses of pediatric septic shock. We tested these hypotheses using a focused analytical approach in which we restricted the working gene list to 296 nuclear-encoded mitochondrial genes, as previously reported by Lunnon *et al.* [14].

## Methods

### Patients and data collection

The study protocol was approved by the Institutional Review Boards of each participating institution: Cincinnati Children’s Hospital Medical Center, The Children’s Hospital of Philadelphia, University of California Benioff Children’s Hospital Oakland, Penn State Hershey Children’s Hospital, Children’s Mercy Hospital, Children’s Hospital of Orange County, Akron Children’s Hospital, Children’s National Medical Center, Miami Children’s Hospital, Texas Children’s Hospital, and CS Mott Children’s Hospital at the University of Michigan. Children ≤10 years of age admitted to the PICU who met pediatric-specific criteria for septic shock were eligible for enrollment [43]. Age-matched controls were recruited from the ambulatory departments of participating institutions using published inclusion and exclusion criteria [36]. All subjects and data collection methods have been previously reported in microarray-based studies addressing hypotheses entirely different from that of the current study and details of the study protocol were previously published [21-23,32,33,36,37,44-48]. All microarray data have been deposited in the National Center for Biotechnology (NCBI) Gene Expression Omnibus (Accession numbers: GSE26440 and GSE26378).

### RNA extraction and microarray hybridization

Written informed consent was obtained from the parents or legal guardians of all septic shock and control subjects to participate in this study. Blood samples were obtained within the first 24 hours of meeting criteria for septic shock. Total RNA was isolated from whole blood using the PaxGene^™^ Blood RNA System (PreAnalytiX, Qiagen/Becton Dickson, Valencia, CA, USA). Microarray hybridization was performed as previously described using the Human Genome U133 Plus 2.0 GeneChip (Affymetrix, Santa Clara, CA, USA) [36].

### Data analysis

We analyzed existing, normalized microarray data. The original analyses were performed using one patient sample per chip. Image files were captured using an Affymetrix GeneChip Scanner 3000. Raw data files (.CEL) were subsequently preprocessed using robust multiple-array average (RMA) normalization and GeneSpring GX 7.3 software (Agilent Technologies, Palo Alto, CA, USA). All signal intensity-based data were used after RMA normalization, which specifically suppresses all but significant variation among lower intensity probe sets [49]. All chips representing septic shock samples were then normalized to the respective median values of controls on a per gene basis.

Differences in mRNA abundance between the study groups were determined using analysis of variance (ANOVA) and corrections for multiple comparisons using a Benjamini-Hochberg false discovery rate (FDR) of 1%. We did not include predetermined fold expression filters in the analysis because the biological implications of a specific threshold change in gene expression are not clear. Thus, in order to account for the possibility that even a modest change in expression within a set of genes from a common metabolic pathway could yield dramatic variability in flux through that pathway [50], we considered all statistical differences in gene expression using the 1% FDR to be significant in this analysis. For clarity, further details regarding microarray data analysis and gene list generation will be provided in the *Results* section.

Gene lists of differentially regulated genes were analyzed using the Ingenuity Pathways Analysis (IPA) application (Ingenuity Systems, Redwood City, CA, USA) to explore potential associations with specific domains of mitochondrial function [44,47,48]. IPA is a database generated from peer-reviewed scientific publications that provides a tool for discovery of signaling pathways and gene networks within the uploaded gene lists. Adjunct analyses of gene lists were conducted using the ToppGene application [51].

Gene expression mosaics representing the expression patterns of differentially regulated genes were generated using the Gene Expression Dynamics Inspector (GEDI) [22,23,52,53]. The signature graphical outputs of GEDI are expression mosaics that give microarray data a ‘face’ that is intuitively recognizable via human pattern recognition. The algorithm for creating the mosaics is a self-organizing map that enables use of human pattern recognition to perform a global analysis of complex genomic data [54].

Ordinal and continuous clinical variables not normally distributed were analyzed via ANOVA on Ranks. Dichotomous clinical variables were analyzed using a chi-square test (SigmaStat Software, Systat Software, Inc., San Jose, CA, USA).

## Results

### Differential regulation of nuclear-encoded mitochondrial genes in patients with septic shock versus healthy controls

One hundred and eighty pediatric subjects with septic shock and 53 healthy pediatric controls were available for analysis. Table 1 provides the demographic characteristics of the two study groups.

**Table 1:** **Subject demographics**

**Variable**	**Controls**	**Septic shock**
	**(n = 53)**	**(n = 180)**
Age, years^a^	2.2 (0.7 - 4.8)	2.4 (0.9 - 6.3)
Male sex, n (%)	31 (58)	109 (61)
Race, n (%)		
Caucasian	33 (62)	119 (66)
African American	12 (23)	35 (19)
Asian	6 (11)	5 (3)
Native Hawaiian or Other Pacific Islander	0 (0)	1 (1)
American Indian/Alaska Native	0 (0)	2 (1)
Multi-racial	1 (2)	4 (2)
Unknown/unreported	1 (2)	14 (8)

^a^Median (interquartile range).

To determine which mitochondrial genes were differentially regulated between patients with septic shock and controls, we conducted an ANOVA starting with 296 nuclear-encoded mitochondrial genes. One hundred and eighteen of the 296 nuclear-encoded mitochondrial genes (40%) were differentially regulated between the two study groups, including 48 upregulated and 70 downregulated in subjects with septic shock. The list of all 118 genes is provided in Table S1 in Additional file 1.

Since limiting the starting gene list for this analysis to the 296 nuclear-encoded mitochondrial genes is a potential source of bias, we conducted an identical analysis starting with all available genes on the array (54,675). This analysis yielded 18,429 differentially regulated genes between septic shock and controls, indicating that the expected rate of differential gene expression is 34% when all genes on the array are considered. Thus, the observed rate of 40% differentially regulated genes when considering only the 296 nuclear-encoded mitochondrial genes is greater than the expected rate of 34% (*P* = 0.025, chi-square). This suggests that our results were not simply due to bias introduced by restricting the analysis to the mitochondrial genes.

**Figure 1:**
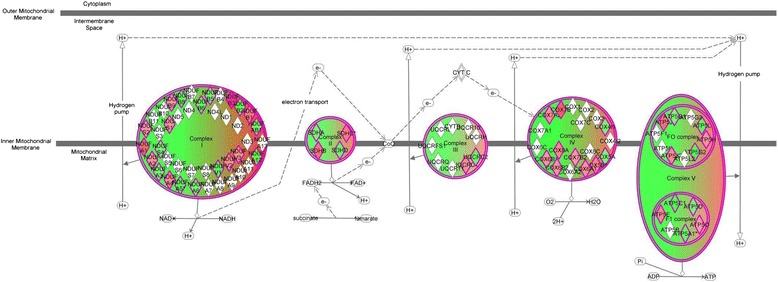
**Differential expression of genes corresponding to the oxidative phosphorylation pathway.** Differential expression of individual nuclear-encoded genes corresponding to mitochondrial electron transport system complexes I to V based on false discovery rate of 1%. Red intensity correlates with increased gene expression and green intensity correlates with decreased gene expression. The 51 genes corresponding to the oxidative phosphorylation pathway were generally downregulated (that is greater degree of green relative to red intensity) in the subjects with septic shock relative to controls.

To visualize the biological function of the 118 differentially regulated genes, we uploaded the gene list to the IPA platform and focused the data output on canonical pathways and gene networks. The top scoring canonical pathway was ‘oxidative phosphorylation’. Figure 1 illustrates the differential regulation of the 51 genes corresponding to the oxidative phosphorylation pathway, which were generally downregulated (that is the degree of green intensity relative to red intensity) in the subjects with septic shock relative to controls (see Table S2 in Additional file 2 for complete gene list).

**Figure 2:**
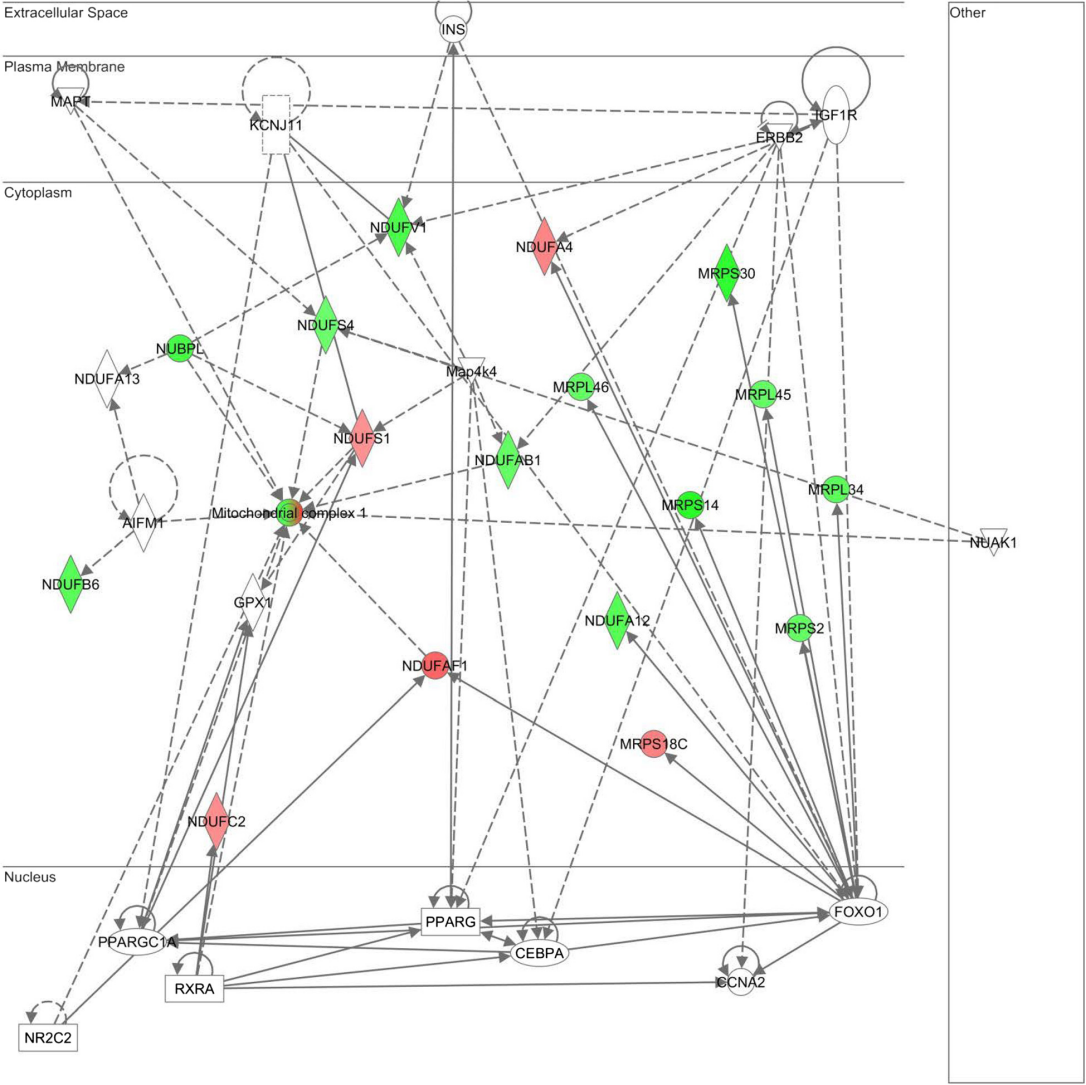
**Differentially regulated genes corresponding to a gene network composed of mitochondrial ribosomal proteins highly connected to the mitochondrial electron transport system (ETS) complex I (nicotinamide adenine dinucleotide (NADH) dehydrogenase).** The degree of green intensity in a gene node corresponds to decreased expression and the degree of red intensity in a given gene node corresponds to increased expression in the subjects with septic shock, relative to controls, respectively. The list of network genes is provided in Table S3 in Additional file 3.

**Figure 3:**
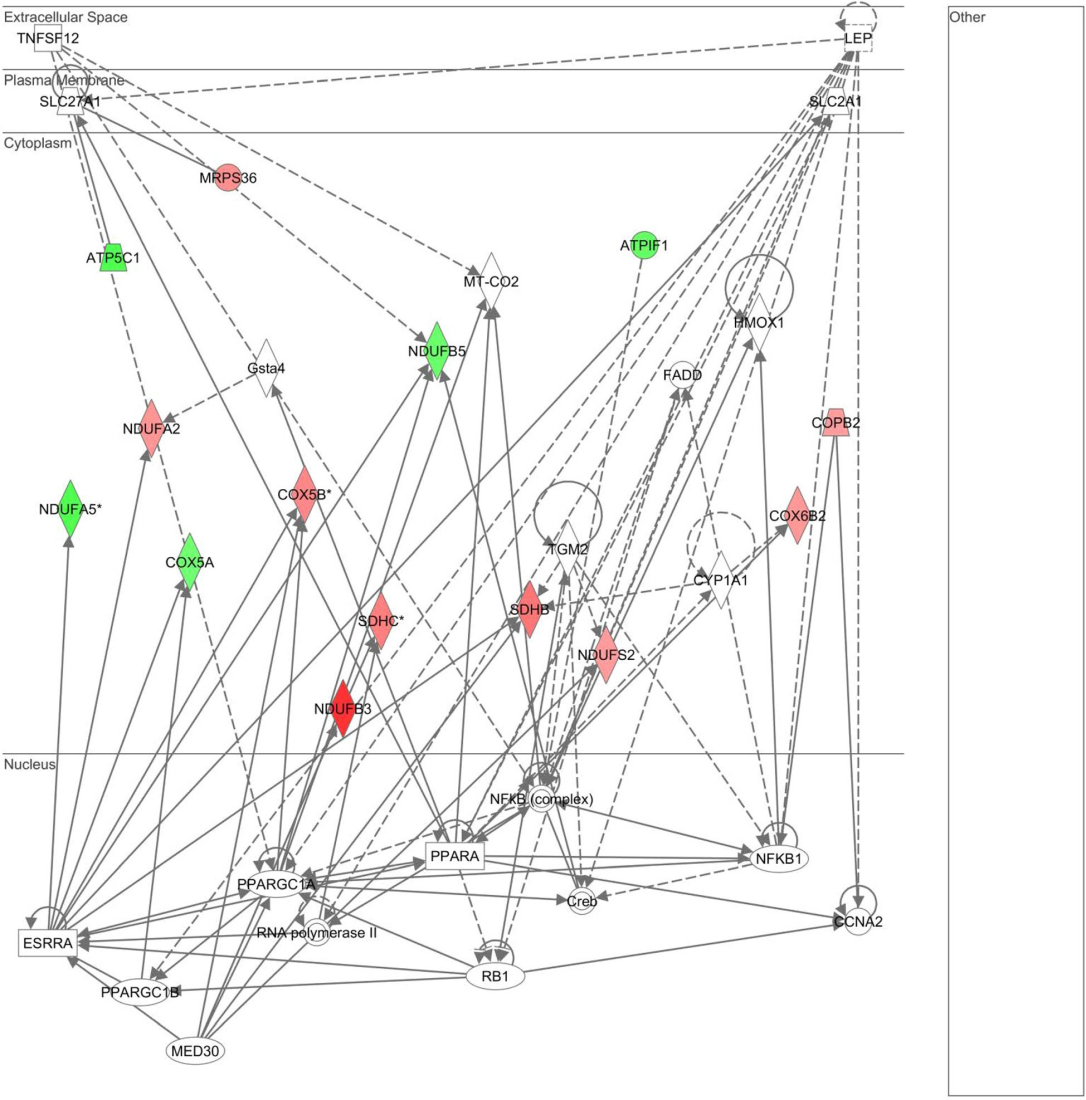
**Differentially regulated genes corresponding to a gene network having peroxisome proliferator-activated receptor (PPAR)-related genes as highly connected nodes.** The degree of green intensity in a gene node corresponds to decreased expression and the degree of red intensity in a given gene node corresponds to increased expression in the subjects with septic shock, relative to controls, respectively. The list of network genes is provided in Table S4 in Additional file 4.

The top two gene networks from the IPA analysis are shown in Figures 2 and 3, with gene nodes being colored based on the degree of increased (red) or decreased (green) expression in subjects with septic shock relative to controls. The gene network shown in Figure 2 is composed primarily of MRPs highly connected to the mitochondrial ETS complex I (nicotinamide adenine dinucleotide (NADH) dehydrogenase), and most of these genes are downregulated in septic shock relative to controls (see Table S3 in Additional file 3 for complete gene list). Consistent with this finding, cross-referencing the network genes to the ToppGene platform returned ‘NADH dehydrogenase activity’ as the top molecular function. The gene network shown in Figure 3 is composed of genes encoding for subunits of ETS complex I, II, and IV that are highly connected to gene nodes corresponding to the peroxisome proliferator-activated receptor (PPAR) family (see Table S4 in Additional file 4 for complete gene list). Cross-referencing the network genes to the ToppGene platform returned the nonspecific function of ‘oxidoreductase activity’ as the top molecular function.

### Differential expression of nuclear-encoded mitochondrial genes across gene expression-based subclasses of pediatric septic shock

We next determined if the 296 nuclear-encoded mitochondrial genes were differentially regulated (ANOVA; 1% FDR) between septic shock survivors (n = 151) and nonsurvivors (n = 29). Two of the 296 nuclear-encoded mitochondrial genes (*cytochrome c oxidase subunit VIIb and NADH dehydrogenase flavoprotein 2, 24 kiloDaltons* (*kDa*)) were differentially regulated between survivors and nonsurvivors. Given the relatively few patients that did not survive, we conducted an alternative analysis in which we compared patients with a ‘complicated course’ (n = 52; defined as patients who either died by 28 days or had persistence of two or more organ failures on day 7 of septic shock) to patients without a complicated course (n = 128) [29,31,55]. Three of the 296 genes (*cytochrome c oxidase subunit VIIb; NADH dehydrogenase flavoprotein 2, 24 kDa; and NADH dehydrogenase 1β subcomplex, 6, 17 kDA*) were differentially regulated between patients with and without a complicated course.

We previously reported and validated three gene expression-based subclasses (groups A, B, and C) of pediatric septic shock having clinically significant phenotypic differences [21-23]. The subclass-defining genes correspond to adaptive immunity, glucocorticoid receptor signaling, and PPARα signaling. Because the gene network shown in Figure 3 contained highly connected gene nodes corresponding to the PPAR family, we next determined if the 296 nuclear-encoded mitochondrial genes were differentially regulated across groups A, B, and C.

The clinical and demographic data for the patients in septic shock groups A (n = 54), B (n = 73), C (n = 53) are shown in Table 2. Patients in group A had a higher mortality rate, pediatric risk of mortality (PRISM) score, pediatric sepsis biomarker risk model (PERSEVERE)-based mortality risk, and maximum number of organ failures compared to patients in groups B and C. Among the patients with available serum lactate data, group A patients had higher median lactate concentrations at study entry compared to groups B and C. There were also some intergroup differences with respect to age, white blood cell counts, and exposure to corticosteroids.

**Table 2:** **Clinical and demographic characteristics of the septic shock subclasses**

**Variable**	**Group A**	**Group B**	**Group C**
	**(n = 54)**	**(n = 73)**	**(n = 53)**
Age, years^a^	1.0 (0.1 - 3.2)	4.5 (1.8 - 8.0)^c^	2.1 (1.2 - 4.8)
Male sex, n (%)	37 (69)	38 (52)	34 (64)
Comorbid condition, n (%)	20 (37)	36 (49)	20 (38)
Blood counts at time of blood draw			
Total WBC count × 10^3^	7.3 (2.4 - 13.6)^d^	15.9 (9.0 - 23.6)	15.0 (7.8 - 19.2)
% Neutrophils	60 (34 - 78)^e^	76 (70 - 85)	72 (61 - 81)
% Lymphocytes	33 (13 - 46)^d^	12 (6 - 22)	18 (9 - 30)
Monocytes	7 (3 - 10)	4 (2 - 8)	6 (3 - 8)
Platelet count × 10^3^	112 (59 - 166)	126 (88 - 228)	183 (67 - 303)
Type of infection, n (%)			
Gram-positive bacteria	19 (36)	18 (25)	15 (28)
Gram-negative bacteria	9 (17)	16 (22)	11 (21)
Other organism	3 (6)	4 (5)	6 (11)
Negative cultures	22 (42)	35 (48)	21 (40)
Maximum number of organ failures^a^	3 (3 - 4)^d^	2 (2 - 3)	2 (2 - 2)
PRISM III score^a^	19 (12 - 31)^d^	12 (9 - 18)	15 (8 - 9)
PERSEVERE mortality probability, %^b^	18.7 (12.4, 25.0)^d^	10.8 (7.1, 14.5)	6.6 (3.2, 10.0)
Insulin, n (%)	1 (2)	5 (7)	2 (4)
Corticosteroids, n (%)	20 (37)	39 (53)	11 (18)^f^
Nonsurvivors, n (%)	16 (30)^d^	9 (12)	4 (8)
Lactate, mmol/L^a^	4.7 (2.4 - 8.3)^g^	1.9 (1.2 - 3.5)	1.7 (0.9 - 2.8)

^a^Median (interquartile range); ^b^mean (95% confidence interval); ^c^ *P* <0.05 compared to groups A and C; ^d^ *P* <0.05 compared to groups B and C; ^e^ *P* <0.05 compared to group B; ^f^ *P* <0.05 compared to groups A and B; ^g^ *P* <0.05 compared to groups B and C. Incomplete data: 28 group A subjects, 45 group B subjects, and 32 group C subjects had available lactate data. PRISM, pediatric risk of mortality; PERSEVERE, pediatric sepsis biomarker risk model.

Using a three-group ANOVA with a false discovery rate of 1%, 162 of the 296 (55%) nuclear-encoded mitochondrial genes were differentially regulated between groups A, B, and C (see Table S5 in Additional file 5 for complete gene list). As a control for this analysis, we generated a random list of 296 genes and conducted an identical three-group ANOVA to determine how many of the randomly selected genes were differentially regulated between groups A, B, and C. We conducted 10 iterations of this process and determined that on average, 118 (± 10 standard deviations) randomly selected genes were differentially regulated between the three groups. Thus, based on random sampling, the expected rate of differentially regulated genes between the three groups is approximately 40%, which is significantly lower than the observed rate of 55% when we focused the analysis to the 296 nuclear-encoded genes (*P* <0.001, chi-square).

**Figure 4:**
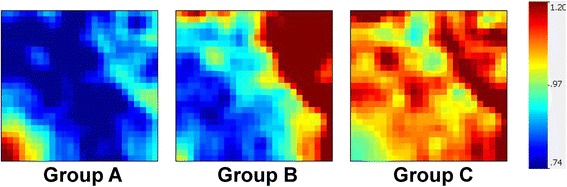
**Gene Expression Dynamics Inspector-generated mosaics of differentially expressed mitochondrial genes for the three septic shock subgroups.** The 162 genes are depicted along the same coordinates across the three expression mosaics. Red intensity correlates with increased gene expression and blue intensity correlates with decreased gene expression. Clear differences in color patterns illustrate differential expression of mitochondrial genes across patient subgroups A, B, and C, with general downregulation in group A. Group A subjects have higher illness severity, higher mortality, and higher organ failure burden.

We next uploaded the values of these 162 genes to the GEDI platform to construct gene expression mosaics for the three septic shock subgroups. Figure 4 shows the respective gene expression mosaics for each subgroup and provides a global representation of how the 162 nuclear-encoded mitochondrial genes were differentially expressed across these three groups. Overall, group A exhibited a greater repression of nuclear-encoded mitochondrial genes, compared to subgroups B and C, as evidenced by the greater proportion of blue color intensity.

**Figure 5:**
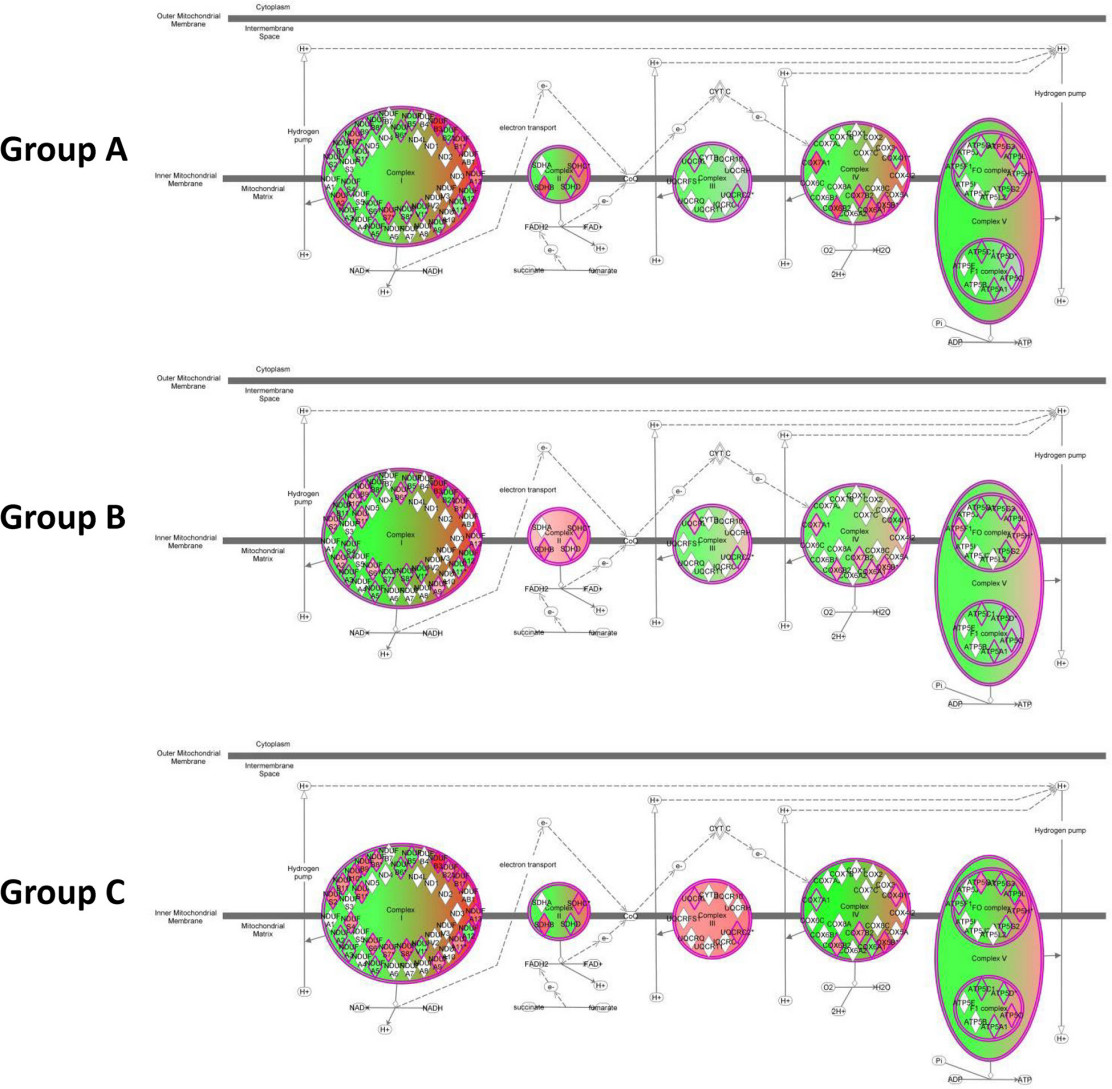
**Differential regulation of oxidative phosphorylation genes for the three septic shock subgroups.** Differential expression of individual nuclear-encoded genes across patient subgroups A, B, and C that correspond to mitochondrial electron transport system (ETS) complexes I to V based on a false discovery rate of 1%. Red intensity correlates with increased gene expression and green intensity correlates with decreased gene expression. Group A exhibited downregulation (that is greater degree of green intensity relative to red intensity) of genes for all ETS complexes, whereas group B exhibited upregulation of genes for complex II and group C exhibited upregulation of genes for complex III.

We next uploaded the 162 mitochondrial gene list to the IPA platform to visualize biological function. This analysis yielded ‘oxidative phosphorylation’ as the most significant canonical pathway rather than a specific domain of mitochondrial function. Figure 5 illustrates the expression of these 47 genes (relative to controls) corresponding to oxidative phosphorylation in each septic shock subclass (see Table S6 in Additional file 6 for complete gene list). Group A exhibited downregulation (that is the degree of green intensity relative to red intensity) of genes for all five ETS complexes. In contrast, group B exhibited upregulation of genes for ETS complex II, with concomitant downregulation of genes for ETS complexes I, III, IV, and V; group C exhibited upregulation of genes for ETS complex III, with concomitant downregulation of genes for ETS complexes I, II, IV, and V.

## Discussion

In this focused analysis of a comprehensive genomic expression database, we found that nuclear-encoded mitochondrial genes are differentially regulated early in pediatric septic shock compared to healthy controls. We also compared expression of the nuclear-encoded mitochondrial genes across previously defined and validated subclasses of pediatric septic shock with distinct phenotypic characteristics. Although these subclasses were defined primarily by differential expression of genes corresponding to adaptive immunity, glucocorticoid receptor signaling, and PPARα signaling, we found that the nuclear-encoded mitochondrial genes were also differentially regulated across these subclasses, with a greater degree of repression in the subclass of patients with the most organ dysfunction and highest mortality.

Fifty-one nuclear genes encoding subunits of the mitochondrial ETS complexes were differentially regulated in blood samples from children with septic shock, with a greater degree of downregulation overall. These findings parallel the decrease in leukocyte gene expression for subunits of the ETS complexes I to V that occurs four to six hours after endotoxin injection in healthy human volunteers [17]. The mitochondrial ETS involves over 100 proteins derived from the nuclear and mitochondrial genomes assembled into five complexes. Although it is difficult to predict how the sum of the changes we observed might alter total function of the respiratory chain, our data raise the notion that genomic influences could affect mitochondrial oxidative respiration and therefore cellular bioenergetic and organ function in sepsis. In particular, the prominence of NADH dehydrogenase activity in the top gene networks is consistent with decreased complex I gene expression [56] and activity noted in skeletal muscle in human adult sepsis [57,58]. Prior studies using blood samples from patients with sepsis have also demonstrated altered respiratory chain activity in peripheral blood mononuclear cells and platelets [9,11,13,59,60].

A similar genome-wide analysis using skeletal muscle from 17 adult patients with sepsis-induced multi-organ failure found that 82 mitochondrial genes were differentially regulated compared to healthy controls (74 upregulated, 8 downregulated) [61]. Decreased ETS enzyme activity was also observed and attributed to a loss of mitochondrial content. The authors concluded that deficits in ETS activity could not be explained by decreased mitochondrial gene expression because of the overall absence of repression of mitochondrial genes. However, these patients were studied later in their septic course when a decrease in mitochondrial content seems to be most prominent, as opposed to our study in which patients were studied in the acute phase when decreased ETS function predominates. More recently, Carre *et al*. performed muscle biopsies early after onset (one to two days) of critical septic illness in adult patients and observed that an overall decrease in ETS gene expression was associated with decreased protein content and activity of ETS complexes I and IV [56]. Concurrent measurements of mitochondrial gene expression and function are needed to determine if the changes we observed in mitochondrial gene expression are sufficient to alter mitochondrial function in blood cells (and other tissues) in pediatric sepsis. We note, however, that serum lactate concentrations were highest in group A. While this suggests a potential association between downregulation of mitochondrial genes and mitochondrial dysfunction, these data should be interpreted cautiously because they are incomplete and represent an indirect, nonspecific measure of mitochondrial function.

Genes encoding the MRPs were also predominately downregulated in pediatric septic shock. This finding should not be construed as specific to mitochondria, as global downregulation of ribosomal gene transcription may occur nonspecifically during extreme biological conditions such as septic shock [17,62]. Similar downregulation of MRPs has been previously described in blood and brain tissue from patients with Alzheimer’s disease [14]. Like bacteria and eukaryotic cell cytoplasm, mitochondria contain their own ribosomes. The MRPs are encoded in nuclear genes, synthesized in the cytoplasm, and then imported into mitochondria. MRPs assemble with mitochondrial-transcribed rRNAs to form two ribosomes that are responsible for translating the 37 mitochondrial-encoded genes, including 13 protein subunits critical to the function of ETS complexes I, III, IV, and V (ATP synthase). Thus, MRPs play a critical role in mitochondrial protein synthesis and bioenergetic function [18,19]. Known mutations in MRPs are associated with lactic acidosis, organ dysfunction, and early death [18], and several MRPs (MRPS29, MRPS30) have been implicated in apoptosis [19]. Moreover, changes in mitochondrial turnover (including biogenesis, mitophagy, and fusion/fission) have been associated with clinical outcomes in sepsis [5,63]. The significant repression of MRPs in our study points to changes in the mitochondrial protein synthesis machinery as one potential mechanism that could lead to mitochondrial dysfunction and diminished mitochondrial content in sepsis.

The differential regulation of mitochondrial genes across subclasses of pediatric septic shock supports a link between mitochondrial gene expression and clinical outcomes, including organ failure and mortality. These subclasses were previously identified based on hierarchical clustering, with patients in group A exhibiting the greatest repression of genes corresponding to key signaling pathways of the adaptive immune system, glucocorticoid receptor signaling, and PPARα signaling [21]. In the current study, patients in group A also exhibited a greater repression of nuclear-encoded mitochondrial genes than groups B and C, especially in ETS complexes II and III. However, we caution that since we observed minimal differential regulation of mitochondrial genes when directly comparing survivors and nonsurvivors, or patients with and without a complicated course, we cannot rule out a ‘coupling effect’ in which changes in mitochondrial gene expression are enhanced by other biologic pathways that differ between genomically defined subclasses. Mitochondria are involved in a variety of cell signaling pathways underlying the immune response, including cytokine release, inflammasome formation, and formation of reactive oxygen species [64]. It will be important to establish the extent to which mitochondrial gene expression may truly affect phenotypic differences in septic shock through its role in the immune system and other cell signaling pathways versus more direct effects on cellular bioenergetics.

We note the limitations of our study. First, this was a *post hoc*, focused analysis using a limited set of 296 nuclear-encoded mitochondrial genes. To reduce the likelihood of false-positive results, we used a relatively stringent FDR of 1% and conducted control analyses to determine expected rates of differential gene expression based on either all available genes on the array, or 10 iterations of analyses based on randomly selected genes. In both cases, we found that the observed rate of differential gene expression in mitochondrial genes was greater than the expected random rate. Nonetheless, our focused analytical approach does not allow us to conclude that the pediatric septic shock transcriptome is specifically enriched for genes corresponding to mitochondrial function. We can only conclude that if the analytical approach is limited to nuclear-encoded mitochondrial genes, we find differential regulation of these genes in children with septic shock, and across subgroups of patients with septic shock.

Second, sufficient data were available from only one time point. Although blood sampling within 24 hours of initial presentation to the PICU with septic shock was likely to capture maximum clinical acuity, we were unable to test temporal changes in the mitochondrial transcriptome with evolution of the septic course. Third, the data are based on whole blood-derived RNA, which carries the potential for confounding by differential white blood cell counts. Although platelet counts did not differ between the three septic shock subclasses, group A had a lower total leukocyte count with a greater percentage of lymphocytes and fewer neutrophils. Lymphocytes have a relatively lower mitochondrial content than neutrophils, though how this effects nuclear-encoded mitochondrial gene expression is not clear [65,66], and have been shown to have slightly less gene upregulation in sepsis [67]. However, we have previously shown that whole blood-derived RNA can yield biologically meaningful data, gene expression profiles have revealed similar themes in leukocyte subsets and whole blood [62], and our current data are consistent with mitochondrial gene expression profiles from previous laboratory- and clinical-based studies [17,56,68-70]. Fourth, since concomitant measures of mitochondrial function were not available we cannot determine how the observed changes might alter the total function of respiratory chain and ATP production. Although the fold change in gene expression was modest in most cases, our findings were similar to magnitude of changes in mitochondrial gene expression profiles observed in prior studies [56,68,71]. However, it also possible that primary mitochondrial bioenergetic dysfunction itself leads to changes in nuclear gene expression [72]. Finally, because we used a nuclear gene array platform, changes in mitochondrial-encoded genes were not included in this study. These genes are critical to the function of the ETS and mitochondrial protein synthesis and should be considered in future studies.

## Conclusions

In summary, our focused analysis demonstrated that nuclear-encoded mitochondrial genes were differentially regulated early in pediatric septic shock compared to healthy controls, as well as across genotypic and phenotypic distinct pediatric septic shock subclasses. Although no pathophysiologic consequences can be derived directly from these results, the findings nonetheless provide support for the hypothesis that differential regulation of nuclear-encoded mitochondrial genes may be an important mechanism contributing to alterations in mitochondrial bioenergetic function in pediatric sepsis.

## Key messages

- Nuclear-encoded mitochondrial genes corresponding to protein subunits of the mitochondrial ETS and mitochondrial ribosomes were differentially expressed in children with septic shock compared to healthy controls.
- Patterns of nuclear-encoded mitochondrial gene expression differed across three previously validated gene expression-based subclasses of pediatric septic shock, with the greatest degree of repression in patients with the most organ dysfunction and highest mortality.
- These findings provide the first evidence that the nuclear genome may be an important mechanism contributing to alterations in mitochondrial bioenergetic function within blood cells in pediatric sepsis.

## Additional files

Additional file 1: Table S1. Mitochondrial genes (n = 118) differentially regulated between subjects with septic shock and normal controls. Additional file 2: Table S2. List of genes (n = 51) corresponding to the oxidative phosphorylation pathway (Figure 1). Additional file 3: Table S3. List of gene nodes in the gene network composed of mitochondrial ribosomal proteins (Figure 2). Additional file 4: Table S4. List of gene nodes in the gene network having peroxisome proliferater-activated receptor (PPAR)-related genes as highly connected nodes (Figure 3). Additional file 5: Table S5. Mitochondrial genes (n = 162) differentially regulated between subjects with septic shock groups A, B, and C. Additional file 6: Table S6. List of genes (n = 47) corresponding to the oxidative phosphorylation pathway and differentially regulated between groups A, B, and C (Figure 5).

## Abbreviations

ANOVA: analysis of variance
ETS: electron transport system
FDR: false discovery rate
GEDI: Gene Expression Dynamics Inspector
IPA: Ingenuity Pathways Analysis
kDa: kiloDaltons
MRP: mitochondrial ribosomal protein
NADH: nicotinamide adenine dinucleotide
NCBI: National Center for Biotechnology
PERSEVERE: pediatric sepsis biomarker risk model
PICU: pediatric intensive care unit
PPAR: peroxisome proliferator-activated receptor
PRISM: pediatric risk of mortality score
RMA: robust multiple-array average
