# Supplementary material for: Differential expression of the nuclear-encoded mitochondrial transcriptome in pediatric septic shock
Source: Crit Care. 2014 Nov 19;18(6):623. doi: 10.1186/s13054-014-0623-9 (PMC4247726; doi:10.1186/s13054-014-0623-9)
Supplement: Additional file 1: Table S1. — Mitochondrial genes (n = 118) differentially regulated between subjects with septic shock and normal controls. [file 13054_2014_623_MOESM1_ESM.doc]

**Additional file 1: Table S1: Mitochondrial genes (n = 118) differentially regulated between subjects with septic shock and normal controls.**

| **Affymetrix ID** | **Fold Change** | **Gene Symbol** | **Description** |
| --- | --- | --- | --- |
| 203371_s_at | 2.348 | NDUFB3 | NADH dehydrogenase (ubiquinone) 1 beta subcomplex, 3, 12kDa |
| 1552485_at | 2.12 | LACTB | lactamase, beta |
| 1552486_s_at | 2.018 | LACTB | lactamase, beta |
| 202110_at | 1.773 | COX7B | cytochrome c oxidase subunit VIIb |
| 204125_at | 1.742 | NDUFAF1 | NADH dehydrogenase (ubiquinone) 1 alpha subcomplex, assembly factor 1 |
| 202298_at | 1.695 | NDUFA1 | NADH dehydrogenase (ubiquinone) 1 alpha subcomplex, 1, 7.5kDa |
| 226354_at | 1.638 | LACTB | lactamase, beta |
| 202675_at | 1.588 | SDHB | succinate dehydrogenase complex, subunit B, iron sulfur (Ip) |
| 202325_s_at | 1.579 | ATP5J | ATP synthase, H+ transporting, mitochondrial F0 complex, subunit F6 |
| 206790_s_at | 1.52 | NDUFB1 | NADH dehydrogenase (ubiquinone) 1 beta subcomplex, 1, 7kDa |
| 203880_at | 1.515 | COX17 | COX17 cytochrome c oxidase assembly homolog (S. cerevisiae) |
| 215088_s_at | 1.475 | SDHC | succinate dehydrogenase complex, subunit C, integral membrane protein, 15kDa |
| 228019_s_at | 1.436 | MRPS18C | mitochondrial ribosomal protein S18C |
| 204599_s_at | 1.412 | MRPL28 | mitochondrial ribosomal protein L28 |
| 210131_x_at | 1.408 | SDHC | succinate dehydrogenase complex, subunit C, integral membrane protein, 15kDa |
| 217773_s_at | 1.392 | NDUFA4 | NADH dehydrogenase (ubiquinone) 1 alpha subcomplex, 4, 9kDa |
| 213736_at | 1.386 | COX5B | Cytochrome c oxidase subunit Vb |
| 218339_at | 1.386 | MRPL22 | mitochondrial ribosomal protein L22 |
| 201119_s_at | 1.379 | COX8A | cytochrome c oxidase subunit 8A (ubiquitous) |
| 202004_x_at | 1.367 | SDHC | succinate dehydrogenase complex, subunit C, integral membrane protein, 15kDa |
| 217801_at | 1.365 | ATP5E | ATP synthase, H+ transporting, mitochondrial F1 complex, epsilon subunit |
| 238056_at | 1.343 | SDHC | succinate dehydrogenase complex, subunit C, integral membrane protein, 15kDa |
| 202961_s_at | 1.302 | ATP5J2 | ATP synthase, H+ transporting, mitochondrial F0 complex, subunit F2 |
| 201597_at | 1.294 | COX7A2 | cytochrome c oxidase subunit VIIa polypeptide 2 (liver) |
| 224302_s_at | 1.287 | MRPS36 | mitochondrial ribosomal protein S36 |
| 217249_x_at | 1.285 | COX7A2 | cytochrome c oxidase subunit VIIa polypeptide 2 (liver) |
| 218101_s_at | 1.284 | NDUFC2 | NADH dehydrogenase (ubiquinone) 1, subcomplex unknown, 2, 14.5kDa |
| 203781_at | 1.279 | MRPL33 | mitochondrial ribosomal protein L33 |
| 226616_s_at | 1.263 | NDUFV3 | NADH dehydrogenase (ubiquinone) flavoprotein 3, 10kDa |
| 211025_x_at | 1.258 | COX5B | cytochrome c oxidase subunit Vb |
| 225523_at | 1.258 | MRPL53 | mitochondrial ribosomal protein L53 |
| 201903_at | 1.254 | UQCRC1 | ubiquinol-cytochrome c reductase core protein I |
| 203039_s_at | 1.24 | NDUFS1 | NADH dehydrogenase (ubiquinone) Fe-S protein 1, 75kDa (NADH-coenzyme Q reductase) |
| 202343_x_at | 1.229 | COX5B | cytochrome c oxidase subunit Vb |
| 209224_s_at | 1.228 | NDUFA2 | NADH dehydrogenase (ubiquinone) 1 alpha subcomplex, 2, 8kDa |
| 210149_s_at | 1.226 | ATP5H | ATP synthase, H+ transporting, mitochondrial F0 complex, subunit d |
| 213735_s_at | 1.216 | COX5B | cytochrome c oxidase subunit Vb |
| 202941_at | 1.205 | NDUFV2 | NADH dehydrogenase (ubiquinone) flavoprotein 2, 24kDa |
| 200925_at | 1.197 | COX6A1 | cytochrome c oxidase subunit VIa polypeptide 1 |
| 1553367_a_at | 1.196 | COX6B2 | cytochrome c oxidase subunit VIb polypeptide 2 (testis) |
| 201441_at | 1.19 | COX6B1 | cytochrome c oxidase subunit Vib polypeptide 1 (ubiquitous) |
| 222992_s_at | 1.185 | NDUFB9 | NADH dehydrogenase (ubiquinone) 1 beta subcomplex, 9, 22kDa |
| 221997_s_at | 1.171 | MRPL52 | Mitochondrial ribosomal protein L52 |
| 201966_at | 1.16 | NDUFS2 | NADH dehydrogenase (ubiquinone) Fe-S protein 2, 49kDa (NADH-coenzyme Q reductase) |
| 226256_at | 1.144 | MRPS22 | mitochondrial ribosomal protein S22 |
| 231265_at | 1.133 | COX7B2 | cytochrome c oxidase subunit VIIb2 |
| 1569891_at | 1.114 | ATP5A1 | ATP synthase, H+ transporting, mitochondrial F1 complex, alpha subunit 1, cardiac muscle |
| 1564482_at | 1.074 | ATP5O | ATP synthase, H+ transporting, mitochondrial F1 complex, O subunit |
| 224869_s_at | 0.899 | MRPS25 | mitochondrial ribosomal protein S25 |
| 208972_s_at | 0.898 | ATP5G1 | ATP synthase, H+ transporting, mitochondrial F0 complex, subunit C1 (subunit 9) |
| 201093_x_at | 0.893 | SDHA | succinate dehydrogenase complex, subunit A, flavoprotein (Fp) |
| 203926_x_at | 0.878 | ATP5D | ATP synthase, H+ transporting, mitochondrial F1 complex, delta subunit |
| 225201_s_at | 0.873 | MRPL14 | mitochondrial ribosomal protein L14 |
| 229426_at | 0.856 | COX5A | cytochrome c oxidase subunit Va |
| 202000_at | 0.855 | NDUFA6 | NADH dehydrogenase (ubiquinone) 1 alpha subcomplex, 6, 14kDa |
| 218563_at | 0.855 | NDUFA3 | NADH dehydrogenase (ubiquinone) 1 alpha subcomplex, 3, 9kDa |
| 222216_s_at | 0.853 | MRPL17 | mitochondrial ribosomal protein L17 |
| 1555057_at | 0.844 | NDUFS4 | NADH dehydrogenase (ubiquinone) Fe-S protein 4, 18kDa (NADH-coenzyme Q reductase) |
| 202698_x_at | 0.84 | COX4I1 | cytochrome c oxidase subunit IV isoform 1 |
| 236910_at | 0.831 | MRPL39 | Mitochondrial ribosomal protein L39 |
| 214330_at | 0.825 | ATPAF2 | ATP synthase mitochondrial F1 complex assembly factor 2 |
| 223743_s_at | 0.825 | MRPL4 | mitochondrial ribosomal protein L4 |
| 203621_at | 0.821 | NDUFB5 | NADH dehydrogenase (ubiquinone) 1 beta subcomplex, 5, 16kDa |
| 224332_s_at | 0.815 | MRPL43 | mitochondrial ribosomal protein L43 |
| 224479_s_at | 0.806 | MRPL45 | mitochondrial ribosomal protein L45 |
| 219244_s_at | 0.801 | MRPL46 | mitochondrial ribosomal protein L46 |
| 202077_at | 0.8 | NDUFAB1 | NADH dehydrogenase (ubiquinone) 1, alpha/beta subcomplex, 1, 8kDa |
| 218001_at | 0.799 | MRPS2 | mitochondrial ribosomal protein S2 |
| 225260_s_at | 0.796 | MRPL32 | mitochondrial ribosomal protein L32 |
| 212600_s_at | 0.794 | UQCRC2 | ubiquinol-cytochrome c reductase core protein II |
| 222775_s_at | 0.794 | MRPL35 | mitochondrial ribosomal protein L35 |
| 215919_s_at | 0.791 | MRPS11 | mitochondrial ribosomal protein S11 |
| 202026_at | 0.79 | SDHD | succinate dehydrogenase complex, subunit D, integral membrane protein |
| 224015_s_at | 0.787 | MRPS25 | mitochondrial ribosomal protein S25 |
| 215850_s_at | 0.784 | NDUFA5 | NADH dehydrogenase (ubiquinone) 1 alpha subcomplex, 5, 13kDa |
| 223339_at | 0.784 | ATPIF1 | ATPase inhibitory factor 1 |
| 218270_at | 0.778 | MRPL24 | mitochondrial ribosomal protein L24 |
| 201717_at | 0.773 | MRPL49 | mitochondrial ribosomal protein L49 |
| 213738_s_at | 0.773 | ATP5A1 | ATP synthase, H+ transporting, mitochondrial F1 complex, alpha subunit 1, cardiac muscle |
| 208907_s_at | 0.772 | MRPS18B | mitochondrial ribosomal protein S18B |
| 224728_at | 0.767 | ATPAF1 | ATP synthase mitochondrial F1 complex assembly factor 1 |
| 212603_at | 0.757 | MRPS31 | mitochondrial ribosomal protein S31 |
| 221692_s_at | 0.755 | MRPL34 | mitochondrial ribosomal protein L34 |
| 225196_s_at | 0.749 | MRPS26 | mitochondrial ribosomal protein S26 |
| 225255_at | 0.748 | MRPL35 | mitochondrial ribosomal protein L35 |
| 224671_at | 0.742 | MRPL10 | mitochondrial ribosomal protein L10 |
| 1559042_at | 0.741 | NDUFB6 | NADH dehydrogenase (ubiquinone) 1 beta subcomplex, 6, 17kDa |
| 218558_s_at | 0.739 | MRPL39 | mitochondrial ribosomal protein L39 |
| 218281_at | 0.737 | MRPL48 | mitochondrial ribosomal protein L48 |
| 218654_s_at | 0.735 | MRPS33 | mitochondrial ribosomal protein S33 |
| 218200_s_at | 0.733 | NDUFB2 | NADH dehydrogenase (ubiquinone) 1 beta subcomplex, 2, 8kDa |
| 208745_at | 0.731 | ATP5L | ATP synthase, H+ transporting, mitochondrial F0 complex, subunit G |
| 225581_s_at | 0.731 | MRPL50 | mitochondrial ribosomal protein L50 |
| 223244_s_at | 0.73 | NDUFA12 | NADH dehydrogenase (ubiquinone) 1 alpha subcomplex, 12 |
| 208822_s_at | 0.715 | DAP3 | death associated protein 3 |
| 211594_s_at | 0.713 | MRPL9 | mitochondrial ribosomal protein L9 |
| 214241_at | 0.71 | NDUFB8 | NADH dehydrogenase (ubiquinone) 1 beta subcomplex, 8, 19kDa |
| 208764_s_at | 0.705 | ATP5G2 | ATP synthase, H+ transporting, mitochondrial F0 complex, subunit C2 (subunit 9) |
| 201304_at | 0.703 | NDUFA5 | NADH dehydrogenase (ubiquinone) 1 alpha subcomplex, 5, 13kDa |
| 226749_at | 0.702 | MRPS9 | mitochondrial ribosomal protein S9 |
| 214132_at | 0.692 | ATP5C1 | ATP synthase, H+ transporting, mitochondrial F1 complex, gamma polypeptide 1 |
| 208714_at | 0.689 | NDUFV1 | NADH dehydrogenase (ubiquinone) flavoprotein 1, 51kDa |
| 217942_at | 0.677 | MRPS35 | mitochondrial ribosomal protein S35 |
| 224948_at | 0.675 | MRPS24 | mitochondrial ribosomal protein S24 |
| 223154_at | 0.662 | MRPL1 | mitochondrial ribosomal protein L1 |
| 220176_at | 0.661 | NUBPL | nucleotide binding protein-like |
| 1558346_at | 0.653 | COX17 | COX17 cytochrome c oxidase assembly homolog (S. cerevisiae) |
| 208787_at | 0.644 | MRPL3 | mitochondrial ribosomal protein L3 |
| 224971_at | 0.62 | MRPL30 | mitochondrial ribosomal protein L30 |
| 209609_s_at | 0.611 | MRPL9 | mitochondrial ribosomal protein L9 |
| 232071_at | 0.604 | MRPL19 | Mitochondrial ribosomal protein L19 |
| 218398_at | 0.598 | MRPS30 | mitochondrial ribosomal protein S30 |
| 227442_at | 0.596 | COX18 | COX18 cytochrome c oxidase assembly homolog (S. cerevisiae) |
| 217408_at | 0.575 | MRPS18B | mitochondrial ribosomal protein S18B |
| 224919_at | 0.575 | MRPS6 | mitochondrial ribosomal protein S6 |
| 203801_at | 0.574 | MRPS14 | mitochondrial ribosomal protein S14 |
| 224873_s_at | 0.571 | MRPS25 | mitochondrial ribosomal protein S25 |
| 212145_at | 0.548 | MRPS27 | mitochondrial ribosomal protein S27 |
