# Supplementary material for: Differential expression of the nuclear-encoded mitochondrial transcriptome in pediatric septic shock
Source: Crit Care. 2014 Nov 19;18(6):623. doi: 10.1186/s13054-014-0623-9 (PMC4247726; doi:10.1186/s13054-014-0623-9)
Supplement: Additional file 2: Table S2. — List of genes (n = 51) corresponding to the oxidative phosphorylation pathway (Figure 1). [file 13054_2014_623_MOESM2_ESM.doc]

**Additional file 2: Table S2: List of genes (n = 51) corresponding to the oxidative phosphorylation pathway (Figure 1).**

| **Gene**  **Symbol** | **Description** |
| --- | --- |
| ATP5A1 | ATP synthase, H+ transporting, mitochondrial F1 complex, alpha subunit 1, cardiac muscle |
| ATP5C1 | ATP synthase, H+ transporting, mitochondrial F1 complex, gamma polypeptide 1 |
| ATP5D | ATP synthase, H+ transporting, mitochondrial F1 complex, delta subunit |
| ATP5E | ATP synthase, H+ transporting, mitochondrial F1 complex, epsilon subunit |
| ATP5G1 | ATP synthase, H+ transporting, mitochondrial Fo complex, subunit C1 (subunit 9) |
| ATP5G2 | ATP synthase, H+ transporting, mitochondrial Fo complex, subunit C2 (subunit 9) |
| ATP5H | ATP synthase, H+ transporting, mitochondrial Fo complex, subunit d |
| ATP5J | ATP synthase, H+ transporting, mitochondrial Fo complex, subunit F6 |
| ATP5J2 | ATP synthase, H+ transporting, mitochondrial Fo complex, subunit F2 |
| ATP5L | ATP synthase, H+ transporting, mitochondrial Fo complex, subunit G |
| ATP5O | ATP synthase, H+ transporting, mitochondrial F1 complex, O subunit |
| ATPAF1 | ATP synthase mitochondrial F1 complex assembly factor 1 |
| ATPAF2 | ATP synthase mitochondrial F1 complex assembly factor 2 |
| COX17 | COX17 cytochrome c oxidase copper chaperone |
| COX4I1 | cytochrome c oxidase subunit IV isoform 1 |
| COX5A | cytochrome c oxidase subunit Va |
| COX5B | cytochrome c oxidase subunit Vb |
| COX6A1 | cytochrome c oxidase subunit VIa polypeptide 1 |
| COX6B1 | cytochrome c oxidase subunit VIb polypeptide 1 (ubiquitous) |
| COX6B2 | cytochrome c oxidase subunit VIb polypeptide 2 (testis) |
| COX7A2 | cytochrome c oxidase subunit VIIa polypeptide 2 (liver) |
| COX7B | cytochrome c oxidase subunit VIIb |
| COX7B2 | cytochrome c oxidase subunit VIIb2 |
| COX8A | cytochrome c oxidase subunit VIIIA (ubiquitous) |
| NDUFA1 | NADH dehydrogenase (ubiquinone) 1 alpha subcomplex, 1, 7.5kDa |
| NDUFA2 | NADH dehydrogenase (ubiquinone) 1 alpha subcomplex, 2, 8kDa |
| NDUFA3 | NADH dehydrogenase (ubiquinone) 1 alpha subcomplex, 3, 9kDa |
| NDUFA4 | NADH dehydrogenase (ubiquinone) 1 alpha subcomplex, 4, 9kDa |
| NDUFA5 | NADH dehydrogenase (ubiquinone) 1 alpha subcomplex, 5 |
| NDUFA6 | NADH dehydrogenase (ubiquinone) 1 alpha subcomplex, 6, 14kDa |
| NDUFA12 | NADH dehydrogenase (ubiquinone) 1 alpha subcomplex, 12 |
| NDUFAB1 | NADH dehydrogenase (ubiquinone) 1, alpha/beta subcomplex, 1, 8kDa |
| NDUFB1 | NADH dehydrogenase (ubiquinone) 1 beta subcomplex, 1, 7kDa |
| NDUFB2 | NADH dehydrogenase (ubiquinone) 1 beta subcomplex, 2, 8kDa |
| NDUFB3 | NADH dehydrogenase (ubiquinone) 1 beta subcomplex, 3, 12kDa |
| NDUFB5 | NADH dehydrogenase (ubiquinone) 1 beta subcomplex, 5, 16kDa |
| NDUFB6 | NADH dehydrogenase (ubiquinone) 1 beta subcomplex, 6, 17kDa |
| NDUFB8 | NADH dehydrogenase (ubiquinone) 1 beta subcomplex, 8, 19kDa |
| NDUFB9 | NADH dehydrogenase (ubiquinone) 1 beta subcomplex, 9, 22kDa |
| NDUFS1 | NADH dehydrogenase (ubiquinone) Fe-S protein 1, 75kDa (NADH-coenzyme Q reductase) |
| NDUFS2 | NADH dehydrogenase (ubiquinone) Fe-S protein 2, 49kDa (NADH-coenzyme Q reductase) |
| NDUFS4 | NADH dehydrogenase (ubiquinone) Fe-S protein 4, 18kDa (NADH-coenzyme Q reductase) |
| NDUFV1 | NADH dehydrogenase (ubiquinone) flavoprotein 1, 51kDa |
| NDUFV2 | NADH dehydrogenase (ubiquinone) flavoprotein 2, 24kDa |
| NDUFV3 | NADH dehydrogenase (ubiquinone) flavoprotein 3, 10kDa |
| SDHA | succinate dehydrogenase complex, subunit A, flavoprotein (Fp) |
| SDHB | succinate dehydrogenase complex, subunit B, iron sulfur (Ip) |
| SDHC | succinate dehydrogenase complex, subunit C, integral membrane protein, 15kDa |
| SDHD | succinate dehydrogenase complex, subunit D, integral membrane protein |
| UQCRC1 | ubiquinol-cytochrome c reductase core protein I |
| UQCRC2 | ubiquinol-cytochrome c reductase core protein II |
