# Supplementary material for: Differential expression of the nuclear-encoded mitochondrial transcriptome in pediatric septic shock
Source: Crit Care. 2014 Nov 19;18(6):623. doi: 10.1186/s13054-014-0623-9 (PMC4247726; doi:10.1186/s13054-014-0623-9)
Supplement: Additional file 3: Table S3. — List of gene nodes in the gene network composed of mitochondrial ribosomal proteins (Figure 2). [file 13054_2014_623_MOESM3_ESM.doc]

**Additional file 3: Table S3: List of gene nodes in the gene network composed of mitochondrial ribosomal proteins (Figure 2) .**

| **Gene Symbol** | **Description** |
| --- | --- |
| AIFM1 | apoptosis-inducing factor, mitochondrion-associated, 1 |
| CCNA2 | cyclin A2 |
| CEBPA | CCAAT/enhancer binding protein (C/EBP), alpha |
| ERBB2 | v-erb-b2 avian erythroblastic leukemia viral oncogene homolog 2 |
| FOXO1 | forkhead box O1 |
| GPX1 | glutathione peroxidase 1 |
| IGF1R | insulin-like growth factor 1 receptor |
| INS | insulin |
| KCNJ11 | potassium inwardly-rectifying channel, subfamily J, member 11 |
| Map4k4 | mitogen-activated protein kinase kinase kinase kinase 4 |
| MAPT | microtubule-associated protein tau |
| MRPL34 | mitochondrial ribosomal protein L34 |
| MRPL45 | mitochondrial ribosomal protein L45 |
| MRPL46 | mitochondrial ribosomal protein L46 |
| MRPS14 | mitochondrial ribosomal protein S14 |
| MRPS18C | mitochondrial ribosomal protein S18C |
| MRPS2 | mitochondrial ribosomal protein S2 |
| MRPS30 | mitochondrial ribosomal protein S30 |
| NDUFA12 | NADH dehydrogenase (ubiquinone) 1 alpha subcomplex, 12 |
| NDUFA13 | NADH dehydrogenase (ubiquinone) 1 alpha subcomplex, 13 |
| NDUFA4 | NADH dehydrogenase (ubiquinone) 1 alpha subcomplex, 4, 9kDa |
| NDUFAB1 | NADH dehydrogenase (ubiquinone) 1, alpha/beta subcomplex, 1, 8kDa |
| NDUFAF1 | NADH dehydrogenase (ubiquinone) complex I, assembly factor 1 |
| NDUFB6 | NADH dehydrogenase (ubiquinone) 1 beta subcomplex, 6, 17kDa |
| NDUFC2 | NADH dehydrogenase (ubiquinone) 1, subcomplex unknown, 2, 14.5kDa |
| NDUFS1 | NADH dehydrogenase (ubiquinone) Fe-S protein 1, 75kDa (NADH-coenzyme Q reductase) |
| NDUFS4 | NADH dehydrogenase (ubiquinone) Fe-S protein 4, 18kDa (NADH-coenzyme Q reductase) |
| NDUFV1 | NADH dehydrogenase (ubiquinone) flavoprotein 1, 51kDa |
| NR2C2 | nuclear receptor subfamily 2, group C, member 2 |
| NUAK1 | NUAK family, SNF1-like kinase, 1 |
| NUBPL | nucleotide binding protein-like |
| PPARG | peroxisome proliferator-activated receptor gamma |
| PPARGC1A | peroxisome proliferator-activated receptor gamma, coactivator 1 alpha |
| RXRA | retinoid X receptor, alpha |
