# Supplementary material for: Differential expression of the nuclear-encoded mitochondrial transcriptome in pediatric septic shock
Source: Crit Care. 2014 Nov 19;18(6):623. doi: 10.1186/s13054-014-0623-9 (PMC4247726; doi:10.1186/s13054-014-0623-9)
Supplement: Additional file 4: Table S4. — List of gene nodes in the gene network having peroxisome proliferater-activated receptor (PPAR)-related genes as highly connected nodes (Figure 3). [file 13054_2014_623_MOESM4_ESM.doc]

**Additional file 4: Table S4: List of gene nodes in the gene network having peroxisome proliferater-activated receptor (PPAR)-related genes as highly connected nodes (Figure 3) .**

| **Gene Symbol** | **Description** |
| --- | --- |
| ATP5C1 | ATP synthase, H+ transporting, mitochondrial F1 complex, gamma polypeptide 1 |
| ATPIF1 | ATPase inhibitory factor 1 |
| CCNA2 | cyclin A2 |
| COPB2 | coatomer protein complex, subunit beta 2 (beta prime) |
| COX5A | cytochrome c oxidase subunit Va |
| COX5B | cytochrome c oxidase subunit Vb |
| COX6B2 | cytochrome c oxidase subunit VIb polypeptide 2 (testis) |
| CYP1A1 | cytochrome P450, family 1, subfamily A, polypeptide 1 |
| ESRRA | estrogen-related receptor alpha |
| FADD | Fas (TNFRSF6)-associated via death domain |
| Gsta4 | glutathione S-transferase, alpha 4 |
| HMOX1 | heme oxygenase (decycling) 1 |
| LEP | leptin |
| MED30 | mediator complex subunit 30 |
| MRPS36 | mitochondrial ribosomal protein S36 |
| MT-CO2 | cytochrome c oxidase subunit II |
| NDUFA2 | NADH dehydrogenase (ubiquinone) 1 alpha subcomplex, 2, 8kDa |
| NDUFA5 | NADH dehydrogenase (ubiquinone) 1 alpha subcomplex, 5 |
| NDUFB3 | NADH dehydrogenase (ubiquinone) 1 beta subcomplex, 3, 12kDa |
| NDUFB5 | NADH dehydrogenase (ubiquinone) 1 beta subcomplex, 5, 16kDa |
| NDUFS2 | NADH dehydrogenase (ubiquinone) Fe-S protein 2, 49kDa (NADH-coenzyme Q reductase) |
| NFKB1 | nuclear factor of kappa light polypeptide gene enhancer in B-cells 1 |
| PPARA | peroxisome proliferator-activated receptor alpha |
| PPARGC1A | peroxisome proliferator-activated receptor gamma, coactivator 1 alpha |
| PPARGC1B | peroxisome proliferator-activated receptor gamma, coactivator 1 beta |
| RB1 | retinoblastoma 1 |
| SDHB | succinate dehydrogenase complex, subunit B, iron sulfur (Ip) |
| SDHC | succinate dehydrogenase complex, subunit C, integral membrane protein, 15kDa |
| SLC27A1 | solute carrier family 27 (fatty acid transporter), member 1 |
| SLC2A1 | solute carrier family 2 (facilitated glucose transporter), member 1 |
| TGM2 | transglutaminase 2 |
| TNFSF12 | tumor necrosis factor (ligand) superfamily, member 12 |
