# Supplementary material for: Differential expression of the nuclear-encoded mitochondrial transcriptome in pediatric septic shock
Source: Crit Care. 2014 Nov 19;18(6):623. doi: 10.1186/s13054-014-0623-9 (PMC4247726; doi:10.1186/s13054-014-0623-9)
Supplement: Additional file 5: Table S5. — Mitochondrial genes (n = 162) differentially regulated between subjects with septic shock groups A, B, and C. [file 13054_2014_623_MOESM5_ESM.doc]

**Additional file 5: Table S5: Mitochondrial genes (n = 162) differentially regulated between subjects with septic shock groups A, B, and C.**

| **Affymetrix ID** | **Gene Symbol** | **Description** |
| --- | --- | --- |
| 203371_s_at | NDUFB3 | NADH dehydrogenase (ubiquinone) 1 beta subcomplex, 3, 12kDa |
| 1553367_a_at | COX6B2 | cytochrome c oxidase subunit VIb polypeptide 2 (testis) |
| 227795_at | NDUFV1 | NADH dehydrogenase (ubiquinone) flavoprotein 1, 51kDa |
| 227024_s_at | MRPL55 | Mitochondrial ribosomal protein L55 |
| 202675_at | SDHB | succinate dehydrogenase complex, subunit B, iron sulfur (Ip) |
| 204570_at | COX7A1 | cytochrome c oxidase subunit VIIa polypeptide 1 (muscle) |
| 1552486_s_at | LACTB | lactamase, beta |
| 209223_at | NDUFA2 | NADH dehydrogenase (ubiquinone) 1 alpha subcomplex, 2, 8kDa |
| 224344_at | COX6A1 | cytochrome c oxidase subunit VIa polypeptide 1 |
| 204125_at | NDUFAF1 | NADH dehydrogenase (ubiquinone) 1 alpha subcomplex, assembly factor 1 |
| 231265_at | COX7B2 | cytochrome c oxidase subunit VIIb2 |
| 242168_at | NDUFS7 | NADH dehydrogenase (ubiquinone) Fe-S protein 7, 20kDa (NADH-coenzyme Q reductase) |
| 226354_at | LACTB | lactamase, beta |
| 234876_at | COX6A1 | cytochrome c oxidase subunit VIa polypeptide 1 |
| 206790_s_at | NDUFB1 | NADH dehydrogenase (ubiquinone) 1 beta subcomplex, 1, 7kDa |
| 1552485_at | LACTB | lactamase, beta |
| 213736_at | COX5B | Cytochrome c oxidase subunit Vb |
| 215088_s_at | SDHC | succinate dehydrogenase complex, subunit C, integral membrane protein, 15kDa |
| 1562392_at | MRPL23 | Mitochondrial ribosomal protein L23 |
| 218225_at | ECSIT | ECSIT homolog (Drosophila) |
| 228019_s_at | MRPS18C | mitochondrial ribosomal protein S18C |
| 218339_at | MRPL22 | mitochondrial ribosomal protein L22 |
| 210131_x_at | SDHC | succinate dehydrogenase complex, subunit C, integral membrane protein, 15kDa |
| 222948_s_at | MRPS18C | mitochondrial ribosomal protein S18C |
| 202004_x_at | SDHC | succinate dehydrogenase complex, subunit C, integral membrane protein, 15kDa |
| 224869_s_at | MRPS25 | mitochondrial ribosomal protein S25 |
| 229165_at | MRPL12 | Mitochondrial ribosomal protein L12 |
| 1554537_at | TMEM126B | transmembrane protein 126B |
| 220864_s_at | NDUFA13 | NADH dehydrogenase (ubiquinone) 1 alpha subcomplex, 13 |
| 239976_at | ACAD9 | Acyl-Coenzyme A dehydrogenase family, member 9 |
| 203931_s_at | MRPL12 | mitochondrial ribosomal protein L12 |
| 238056_at | SDHC | succinate dehydrogenase complex, subunit C, integral membrane protein, 15kDa |
| 228301_x_at | NDUFB10 | NADH dehydrogenase (ubiquinone) 1 beta subcomplex, 10, 22kDa |
| 221997_s_at | MRPL52 | Mitochondrial ribosomal protein L52 |
| 211025_x_at | COX5B | cytochrome c oxidase subunit Vb |
| 210008_s_at | MRPS12 | mitochondrial ribosomal protein S12 |
| 228689_at | NDUFA11 | NADH dehydrogenase (ubiquinone) 1 alpha subcomplex, 11, 14.7kDa |
| 218887_at | MRPL2 | mitochondrial ribosomal protein L2 |
| 218049_s_at | MRPL13 | mitochondrial ribosomal protein L13 |
| 228168_at | ATP5G3 | ATP synthase, H+ transporting, mitochondrial F0 complex, subunit C3 (subunit 9) |
| 228355_s_at | NDUFAF2 | NADH dehydrogenase (ubiquinone) 1 alpha subcomplex, assembly factor 2 |
| 218105_s_at | MRPL4 | mitochondrial ribosomal protein L4 |
| 222993_at | MRPL37 | mitochondrial ribosomal protein L37 |
| 225523_at | MRPL53 | mitochondrial ribosomal protein L53 |
| 210149_s_at | ATP5H | ATP synthase, H+ transporting, mitochondrial F0 complex, subunit d |
| 218101_s_at | NDUFC2 | NADH dehydrogenase (ubiquinone) 1, subcomplex unknown, 2, 14.5kDa |
| 225425_s_at | MRPL41 | mitochondrial ribosomal protein L41 |
| 226256_at | MRPS22 | mitochondrial ribosomal protein S22 |
| 203781_at | MRPL33 | mitochondrial ribosomal protein L33 |
| 222992_s_at | NDUFB9 | NADH dehydrogenase (ubiquinone) 1 beta subcomplex, 9, 22kDa |
| 225201_s_at | MRPL14 | mitochondrial ribosomal protein L14 |
| 223112_s_at | NDUFB10 | NADH dehydrogenase (ubiquinone) 1 beta subcomplex, 10, 22kDa |
| 203926_x_at | ATP5D | ATP synthase, H+ transporting, mitochondrial F1 complex, delta subunit |
| 218112_at | MRPS34 | mitochondrial ribosomal protein S34 |
| 225719_s_at | MRPL55 | mitochondrial ribosomal protein L55 |
| 224302_s_at | MRPS36 | mitochondrial ribosomal protein S36 |
| 201966_at | NDUFS2 | NADH dehydrogenase (ubiquinone) Fe-S protein 2, 49kDa (NADH-coenzyme Q reductase) |
| 203039_s_at | NDUFS1 | NADH dehydrogenase (ubiquinone) Fe-S protein 1, 75kDa (NADH-coenzyme Q reductase) |
| 224247_s_at | MRPS10 | mitochondrial ribosomal protein S10 |
| 211595_s_at | MRPS11 | mitochondrial ribosomal protein S11 |
| 226241_s_at | MRPL52 | mitochondrial ribosomal protein L52 |
| 236779_at | MRPS5 | Mitochondrial ribosomal protein S5 |
| 1555998_at | ATP5H | ATP synthase, H+ transporting, mitochondrial F0 complex, subunit d |
| 225103_at | MRPL38 | mitochondrial ribosomal protein L38 |
| 218202_x_at | MRPL44 | mitochondrial ribosomal protein L44 |
| 208972_s_at | ATP5G1 | ATP synthase, H+ transporting, mitochondrial F0 complex, subunit C1 (subunit 9) |
| 228059_x_at | MRPS22 | mitochondrial ribosomal protein S22 |
| 222466_s_at | MRPL42 | mitochondrial ribosomal protein L42 |
| 223448_x_at | MRPS22 | mitochondrial ribosomal protein S22 |
| 223292_s_at | MRPS15 | mitochondrial ribosomal protein S15 |
| 226296_s_at | MRPS15 | mitochondrial ribosomal protein S15 |
| 220103_s_at | MRPS18C | mitochondrial ribosomal protein S18C |
| 203189_s_at | NDUFS8 | NADH dehydrogenase (ubiquinone) Fe-S protein 8, 23kDa (NADH-coenzyme Q reductase) |
| 223742_at | MRPL4 | mitochondrial ribosomal protein L4 |
| 218563_at | NDUFA3 | NADH dehydrogenase (ubiquinone) 1 alpha subcomplex, 3, 9kDa |
| 222499_at | MRPS16 | mitochondrial ribosomal protein S16 |
| 232169_x_at | NDUFS8 | NADH dehydrogenase (ubiquinone) Fe-S protein 8, 23kDa (NADH-coenzyme Q reductase) |
| 222555_s_at | MRPL44 | mitochondrial ribosomal protein L44 |
| 207618_s_at | BCS1L | BCS1-like (yeast) |
| 209065_at | UQCRB | ubiquinol-cytochrome c reductase binding protein |
| 211752_s_at | NDUFS7 | NADH dehydrogenase (ubiquinone) Fe-S protein 7, 20kDa (NADH-coenzyme Q reductase) |
| 240391_at | NDUFB2 | NADH dehydrogenase (ubiquinone) 1 beta subcomplex, 2, 8kDa |
| 224331_s_at | MRPL36 | mitochondrial ribosomal protein L36 |
| 203800_s_at | MRPS14 | mitochondrial ribosomal protein S14 |
| 236356_at | NDUFS1 | NADH dehydrogenase (ubiquinone) Fe-S protein 1, 75kDa (NADH-coenzyme Q reductase) |
| 223221_at | SCO1 | SCO cytochrome oxidase deficient homolog 1 (yeast) |
| 228690_s_at | NDUFA11 | NADH dehydrogenase (ubiquinone) 1 alpha subcomplex, 11, 14.7kDa |
| 243630_at | NDUFB1 | NADH dehydrogenase (ubiquinone) 1 beta subcomplex, 1, 7kDa |
| 218270_at | MRPL24 | mitochondrial ribosomal protein L24 |
| 203606_at | NDUFS6 | NADH dehydrogenase (ubiquinone) Fe-S protein 6, 13kDa (NADH-coenzyme Q reductase) |
| 216954_x_at | ATP5O | ATP synthase, H+ transporting, mitochondrial F1 complex, O subunit |
| 226257_x_at | MRPS22 | mitochondrial ribosomal protein S22 |
| 204295_at | SURF1 | surfeit 1 |
| 227186_s_at | MRPL41 | mitochondrial ribosomal protein L41 |
| 218890_x_at | MRPL35 | mitochondrial ribosomal protein L35 |
| 205241_at | SCO2 | SCO cytochrome oxidase deficient homolog 2 (yeast) |
| 224160_s_at | ACAD9 | acyl-Coenzyme A dehydrogenase family, member 9 |
| 217368_at | ATP5G2 | ATP synthase, H+ transporting, mitochondrial F0 complex, subunit C2 (subunit 9) |
| 208969_at | NDUFA9 | NADH dehydrogenase (ubiquinone) 1 alpha subcomplex, 9, 39kDa |
| 201227_s_at | NDUFB8 | NADH dehydrogenase (ubiquinone) 1 beta subcomplex, 8, 19kDa |
| 223743_s_at | MRPL4 | mitochondrial ribosomal protein L4 |
| 219220_x_at | MRPS22 | mitochondrial ribosomal protein S22 |
| 237560_at | MRPS5 | Mitochondrial ribosomal protein S5 |
| 230027_s_at | MRPL43 | mitochondrial ribosomal protein L43 |
| 203613_s_at | NDUFB6 | NADH dehydrogenase (ubiquinone) 1 beta subcomplex, 6, 17kDa |
| 211755_s_at | ATP5F1 | ATP synthase, H+ transporting, mitochondrial F0 complex, subunit B1 |
| 204331_s_at | MRPS12 | mitochondrial ribosomal protein S12 |
| 203190_at | NDUFS8 | NADH dehydrogenase (ubiquinone) Fe-S protein 8, 23kDa (NADH-coenzyme Q reductase) |
| 217919_s_at | MRPL42 | mitochondrial ribosomal protein L42 |
| 218320_s_at | NDUFB11 | NADH dehydrogenase (ubiquinone) 1 beta subcomplex, 11, 17.3kDa |
| 213041_s_at | ATP5D | ATP synthase, H+ transporting, mitochondrial F1 complex, delta subunit |
| 213758_at | COX4I1 | cytochrome c oxidase subunit IV isoform 1 |
| 208907_s_at | MRPS18B | mitochondrial ribosomal protein S18B |
| 243821_at | MRPS31 | mitochondrial ribosomal protein S31 |
| 223338_s_at | ATPIF1 | ATPase inhibitory factor 1 |
| 217646_at | SURF1 | surfeit 1 |
| 1555057_at | NDUFS4 | NADH dehydrogenase (ubiquinone) Fe-S protein 4, 18kDa (NADH-coenzyme Q reductase) |
| 219244_s_at | MRPL46 | mitochondrial ribosomal protein L46 |
| 221622_s_at | TMEM126B | transmembrane protein 126B |
| 218001_at | MRPS2 | mitochondrial ribosomal protein S2 |
| 200086_s_at | COX4I1 | cytochrome c oxidase subunit IV isoform 1 |
| 224015_s_at | MRPS25 | mitochondrial ribosomal protein S25 |
| 224729_s_at | ATPAF1 | ATP synthase mitochondrial F1 complex assembly factor 1 |
| 202698_x_at | COX4I1 | cytochrome c oxidase subunit IV isoform 1 |
| 212604_at | MRPS31 | mitochondrial ribosomal protein S31 |
| 224173_s_at | MRPL30 | mitochondrial ribosomal protein L30 |
| 241755_at | UQCRC2 | ubiquinol-cytochrome c reductase core protein II |
| 203621_at | NDUFB5 | NADH dehydrogenase (ubiquinone) 1 beta subcomplex, 5, 16kDa |
| 224479_s_at | MRPL45 | mitochondrial ribosomal protein L45 |
| 200883_at | UQCRC2 | ubiquinol-cytochrome c reductase core protein II |
| 215919_s_at | MRPS11 | mitochondrial ribosomal protein S11 |
| 218106_s_at | MRPS10 | mitochondrial ribosomal protein S10 |
| 212600_s_at | UQCRC2 | ubiquinol-cytochrome c reductase core protein II |
| 208714_at | NDUFV1 | NADH dehydrogenase (ubiquinone) flavoprotein 1, 51kDa |
| 235533_at | COX19 | COX19 cytochrome c oxidase assembly homolog (S. cerevisiae) |
| 224728_at | ATPAF1 | ATP synthase mitochondrial F1 complex assembly factor 1 |
| 224948_at | MRPS24 | mitochondrial ribosomal protein S24 |
| 218200_s_at | NDUFB2 | NADH dehydrogenase (ubiquinone) 1 beta subcomplex, 2, 8kDa |
| 223244_s_at | NDUFA12 | NADH dehydrogenase (ubiquinone) 1 alpha subcomplex, 12 |
| 215850_s_at | NDUFA5 | NADH dehydrogenase (ubiquinone) 1 alpha subcomplex, 5, 13kDa |
| 221692_s_at | MRPL34 | mitochondrial ribosomal protein L34 |
| 203465_at | MRPL19 | mitochondrial ribosomal protein L19 |
| 214241_at | NDUFB8 | NADH dehydrogenase (ubiquinone) 1 beta subcomplex, 8, 19kDa |
| 223339_at | ATPIF1 | ATPase inhibitory factor 1 |
| 208745_at | ATP5L | ATP synthase, H+ transporting, mitochondrial F0 complex, subunit G |
| 224671_at | MRPL10 | mitochondrial ribosomal protein L10 |
| 208822_s_at | DAP3 | death associated protein 3 |
| 213738_s_at | ATP5A1 | ATP synthase, H+ transporting, mitochondrial F1 complex, alpha subunit 1, cardiac muscle |
| 202026_at | SDHD | succinate dehydrogenase complex, subunit D, integral membrane protein |
| 1558346_at | COX17 | COX17 cytochrome c oxidase assembly homolog (S. cerevisiae) |
| 1559042_at | NDUFB6 | NADH dehydrogenase (ubiquinone) 1 beta subcomplex, 6, 17kDa |
| 208764_s_at | ATP5G2 | ATP synthase, H+ transporting, mitochondrial F0 complex, subunit C2 (subunit 9) |
| 212603_at | MRPS31 | mitochondrial ribosomal protein S31 |
| 209609_s_at | MRPL9 | mitochondrial ribosomal protein L9 |
| 214132_at | ATP5C1 | ATP synthase, H+ transporting, mitochondrial F1 complex, gamma polypeptide 1 |
| 224971_at | MRPL30 | mitochondrial ribosomal protein L30 |
| 218398_at | MRPS30 | mitochondrial ribosomal protein S30 |
| 227442_at | COX18 | COX18 cytochrome c oxidase assembly homolog (S. cerevisiae) |
| 217408_at | MRPS18B | mitochondrial ribosomal protein S18B |
| 212145_at | MRPS27 | mitochondrial ribosomal protein S27 |
| 224919_at | MRPS6 | mitochondrial ribosomal protein S6 |
| 232071_at | MRPL19 | Mitochondrial ribosomal protein L19 |
